# High-Mobility Electrons in Aqueous Iodide Solutions

**DOI:** 10.1021/acsomega.4c11040

**Published:** 2025-02-02

**Authors:** Fabio Novelli, Adrian Buchmann, Iqra Yousaf, Lion-Luca Stiewe, Wibke Bronsch, Federico Cilento, Claudius Hoberg, Martina Havenith

**Affiliations:** 1 Department of Physical Chemistry II, Ruhr University Bochum, Bochum 44801, Germany; 2 Elettra - Sincrotrone Trieste S.C.p.A., Strada Statale 14, km 163.5, Trieste I-34149, Italy

## Abstract

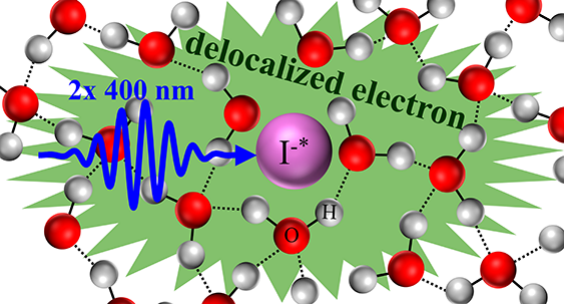

The photoexcitation of aqueous iodide solutions is a prototype for the generation of electrons in liquid water. Upon one-photon excitation, the precursors of the solvated electrons are localized states with a radius of a few angstroms. In contrast, with the aid of transient absorption spectroscopy at terahertz, near-infrared, and visible frequencies, we show that the two-photon absorption of ∼400 nm pulses can impulsively generate short-lived (∼250 fs), delocalized electrons that are released tens of angstroms away from the parent ion. We propose that these states can be ascribed to 5p → 6p transitions that, in turn, could be thought of as frustrated Rydberg orbitals or large radius excitons. By capitalizing on the unique capabilities of transient terahertz spectroscopy, we estimate that these delocalized states are characterized by an electronic mobility and diffusivity that are about 500 times greater than those of the fully relaxed electrons.

## Introduction

Aqueous iodide displays two features centered at ∼5.5 eV (226 nm) and ∼6.4 eV (193 nm) in the linear or one-photon absorption spectrum that are assigned to charge transfer to solvent (CTTS) transitions from the 5p to the 6s atomic orbital of the anion.^1−10^ Model calculations suggest that the resonant excitation of these transitions ejects electrons to a short distance,^1,5,11^ ∼3–8 Å, within^1,4,11^ ∼100–250 fs. This results in the formation of a contact pair (CP) of the photogenerated iodine atom and an unrelaxed localized electron that, in turn, decays on a ∼1 ps time scale^1,4^ and forms a solvent-separated (SS) electron–ion pair. The short ejection length limits the lifetime of the electrons, which recombine with the parent ion within^1^ 6 ps. This picture is corroborated by time-resolved photoemission and transient absorption spectroscopic experiments^2,3,15,16,10,12−14^ that reported time constants between 200 and 400 fs for the formation of the CP,^3,6,8,10,12,15^ 0.7 and 1.7 ps for the SS pair,^8,12,15^ and 15 to 33 ps for the geminate recombination.^3,6,8,10,12,15,16^

The three-photon absorption of iodide solutions was studied before at near-infrared (NIR) and visible (VIS) frequencies.^13,14^ These results resemble the ones found in pure water when the total absorbed energy exceeds^17−21^ ∼9.3 eV: the photogenerated electrons are impulsively delocalized and ejected to distances up to^22−24^ 20–40 Å. These “free” electrons (e_free_) decay within^22−24^ ∼200 fs into an intermediate state with a radius of^25^ 4–5 Å that is called weakly bound, quasi-bound, presolvated, or “wet” electron (e_wet_).^26,27^ The wet electron converts into the aqueous, solvated electron (e_solv_) with a characteristic time constant of^25,28,29^ 300–600 fs. On a time scale of 400–1200 fs, the solvated electron collapses forming a cavity with a 2–3 Å radius,^30,31^ surrounded by 4–6 water molecules that have weaker hydrogen bonds with respect to a molecule in the bulk liquid phase.^22,25,29^ The solvated electron recombines on a time scale exceeding several hundred picoseconds.^32−36^

We showed in previous optical-pump terahertz-probe (OPTP) measurements that the absorption of two ∼3.1 eV (400 nm) photons by aqueous iodide solutions can result in a large (∼20%) and fast (∼70 fs) modulation of the transient response at terahertz (THz) frequencies.^37^ In order to clarify the microscopic origin of this signal,^38,39^ here we report a combined study with probe pulses in the THz, NIR, and VIS frequency ranges. Based on our results, we propose that the large THz modulation can be ascribed to the generation of impulsively delocalized electrons with a lifetime of ∼250 fs and a mobility of ∼0.9 cm^2^/(V·s).

## Methods

We prepared a series of concentrated aqueous solutions of sodium iodide (>99.5% NaI, s3) by dissolving the appropriate weighted amount of salt in ultrapure, milli-Q liquid water. The concentration is measured in moles (mol/L = M). To avoid possible pump–probe signals originating from the sample holder windows, we used a free-flowing flat-jet with a width of about 1 cm and a thickness of ∼10 μm. The flat-jet system was previously characterized in ref..^40^ The liquid reservoir was kept at room temperature (RT), 20.0 ± 0.1°*C*.

**Figure 1:**
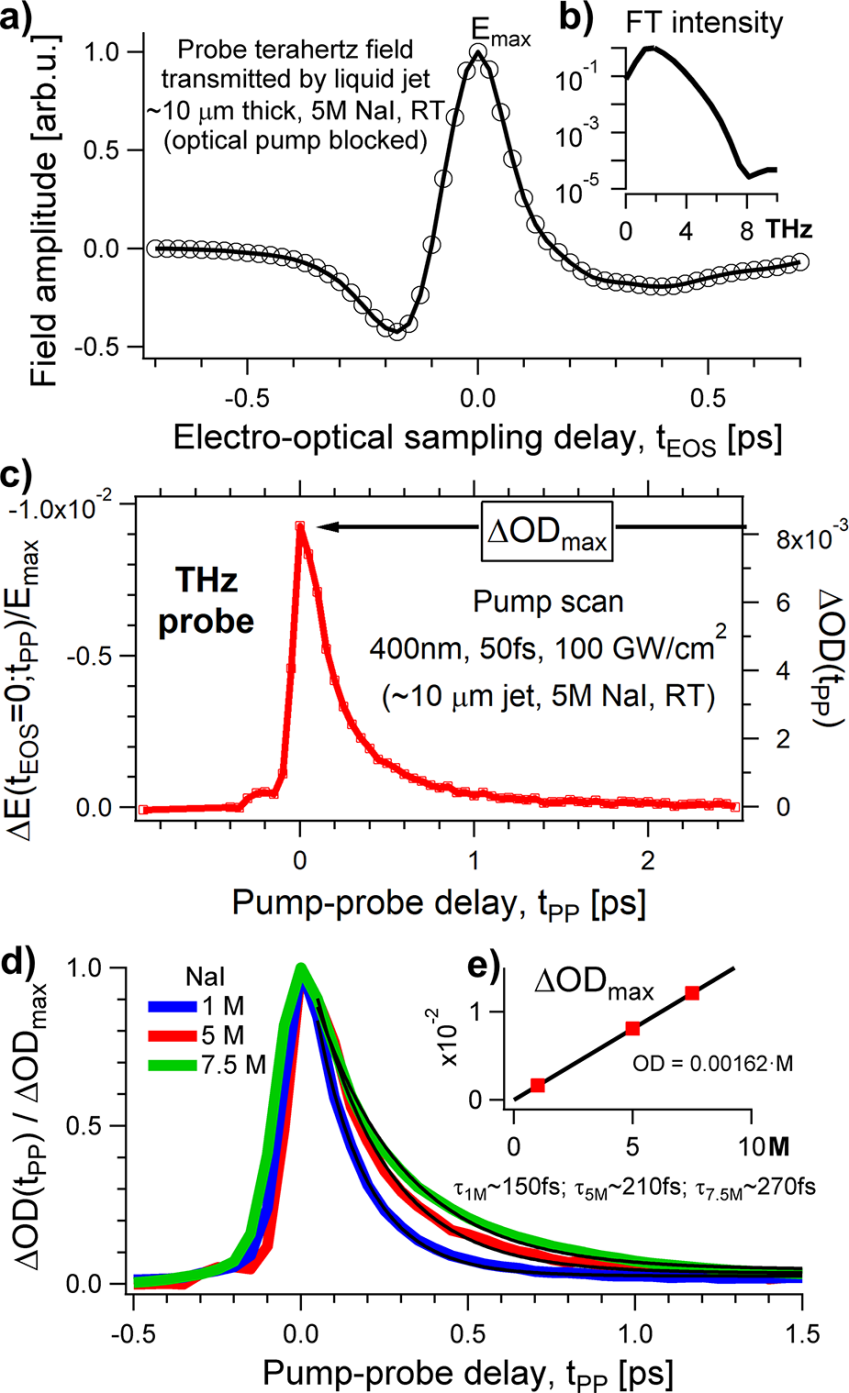
Optical-pump terahertz-probe results. a) THz pulse transmitted at equilibrium, with the optical pump off, by a free-flowing 10-μm thick aqueous solution containing 5 M of sodium iodide. The position of the THz peak is marked with *E*_max_. b) Intensity (magnitude squared) of the Fourier transform of the THz field. c) Relative variation of the transmitted THz peak, Δ*E*/*E*_max_, as a function of the pump–probe delay, t_PP_. The pump pulses are centered at about 400 nm, are 50 fs long, and have an intensity of ∼100 GW/cm^2^. The right axis refers to the corresponding change in the optical density, ΔOD(t_PP_). d) Normalized transient OD as a function of the salt concentration: 1 M in blue, 5 M in red, and 7.5 M NaI in green. The decay time increases with salt concentration, from ∼150 fs for 1 M to ∼270 fs for 7.5M. e) The maximum change of the optical density is found at pump–probe overlap (t_PP_ = 0) and scales linearly with the salt concentration.

At first, we used the in-house OPTP optical setup. The broadband terahertz pulses are generated via two-color plasma filamentation in nitrogen of ∼50 fs long pulses centered at ∼800 nm. The THz fields were detected in a 100 μm thick gallium phosphide (GaP) crystal by scanning the delay of an electro-optical sampling beam, t_EOS_. The field transmitted by the 5 M NaI aqueous jet is shown in Figure 1a. The maximum THz field is transmitted at t_EOS_ = 0 and marked with *E*_max_. Figure 1b shows the spectrum, which is equal to the squared magnitude of the Fourier transform of the THz pulse. This spectrum extends up to ∼8 THz. We established before^22^ that ∼1.5 THz could be selected as the lowest reliable frequency, as determined by the acquisition range of the sampling delay and possible diffraction effects. Considering that the intensity drops to about 1% at 5.5 THz, we restrict further discussions to the 1.5–5.5 THz frequency range. We performed “pump scan” experiments whereby the sampling time is fixed to the position of the peak THz field (t_EOS_ = 0) and the optical pulse is delayed.^41^ As the frequency components of the THz pulse add up constructively at the peak position, the transient variation of *E*_max_ corresponds to the response of the sample integrated over the available THz spectrum. Further details about the OPTP setup can be found in ref.^42,43^.

In addition, we performed transient absorption experiments with visible and near-infrared probe pulses at the T-ReX laser facility located at the FERMI free-electron laser in Trieste. The duration of the pulses emitted by this source amounts to ∼100 fs. We obtained the probe pulses via supercontinuum generation in a sapphire plate.^44^ As reported for example in ref.^45^, the full spectral response could not be reliably recorded due to an artifact in the white light probe around the seed wavelength of ∼800 nm. In previous investigations, Long et al.^13,14^ chose 625 and 1000 nm as the optimal wavelengths to probe the dynamics of the electronic states in aqueous iodide, while Wang et al.^26^ highlighted the transient response at 600 and 1200 nm. For the experiments reported here, we selected two significant probe wavelengths that are sufficiently distant from the seed input, at λ∼1000 nm in the NIR and λ∼600 nm in the VIS.

The probe pulses had a repetition rate of 1 kHz in all measurements. The arrival time of the pump was varied with a mechanical stage, determining the pump–probe delay (t_PP_). The pump beam was chopped at 500 Hz, set to a central wavelength of ∼400 nm via second harmonic generation, and had a tunable peak intensity reaching ∼100 GW/cm^2^.

## Results

In the OPTP experiments, the relative change of the THz peak transmission, , was detected as a function of the pump–probe delay. The pump–probe signal for the 5 M iodide solution is plotted in Figure 1c. From the relative variation of the peak field, it is possible to estimate the optical density (OD) of the material as a function of the pump-probe delay. . The OD scale is plotted on the right axis of Figure 1c. The maximum value, ΔO*D*_max_, is obtained close to pulse overlap (t_PP_ = 0) and amounts to 8.1 ± 0.1 *mOD*, where ±0.1 *mOD* is the typical noise level of the OPTP measurements.

We report in Figure 1d the normalized quantity  for different concentrations of NaI. The blue, red, and green curves show the OPTP response for a 1M, 5M, and 7.5 M NaI aqueous solution, respectively. The transient signals fit an exponential function *y* ∝ *e*^–(*t*–*t*_0_)/τ_*C*_^, where t_0_ = 50 fs is the fixed initial time point and τ_C_ is the relaxation time-constant for the aqueous solution containing a concentration *C* of iodide salt. The exponential fit functions are shown with the solid black lines in Figure 1d. The relaxation time-constant increases with concentration: τ_1M_ ∼ 150 fs, τ_5M_ ∼ 210 fs, and τ_7.5M_ ∼ 270 fs. While the statistical errors of the fit are smaller than 10 fs, we estimate a systematic uncertainty of half the pulse duration, ± 25 *fs*. In Figure 1e we show that the maximum signal at t_PP_ = 0 is proportional to the salt concentration, see the black line.

**Figure 2:**
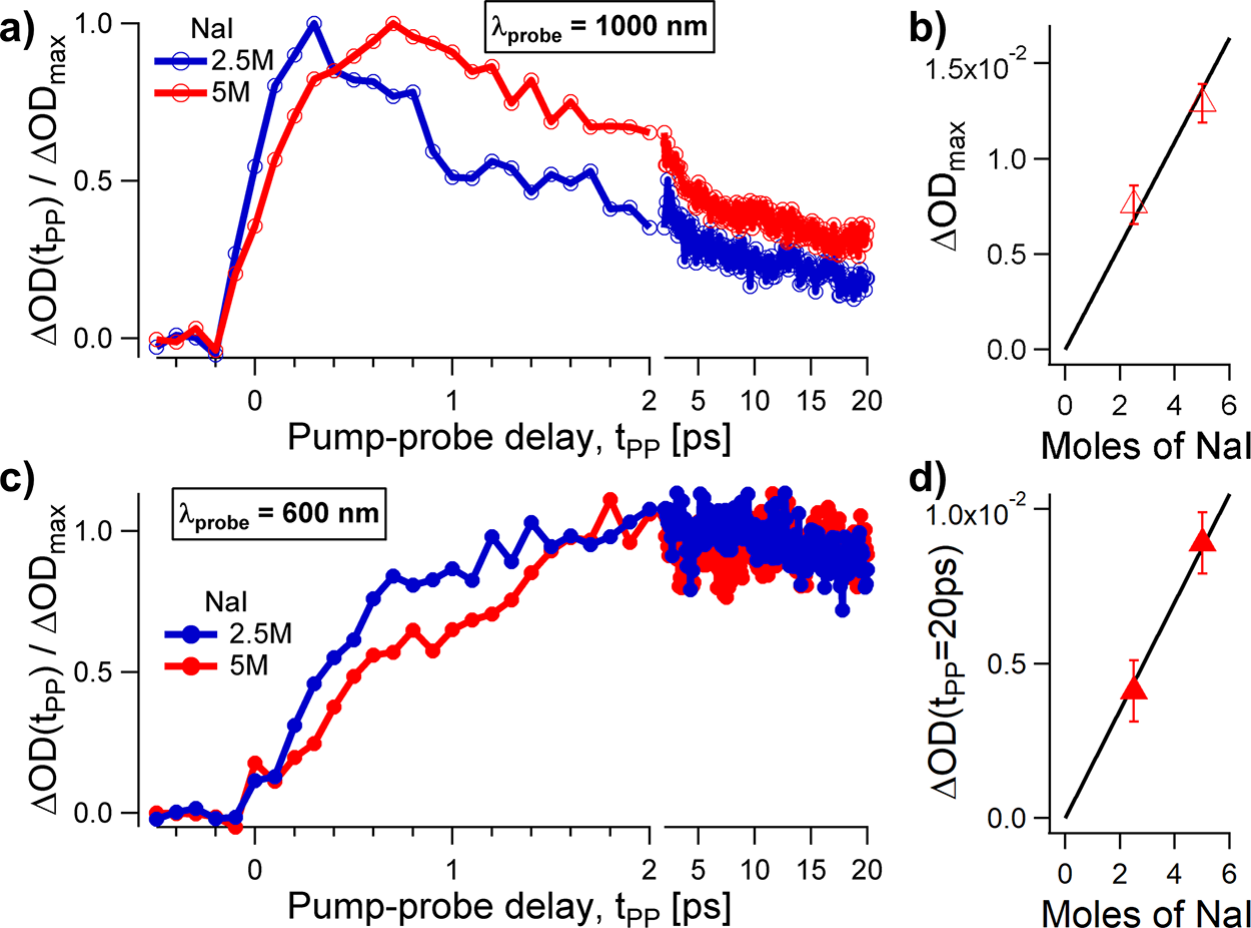
Transient absorption probed at infrared (a,b) and visible (c,d) wavelengths. The ∼10 μm thick liquid jet is irradiated with 400 nm, 100 fs long pulses with a peak intensity of ∼100 GW/cm^2^. a) When the central probe wavelength is set to λ = 1000 nm, the signal increases within 100–300 fs and decays within about 1 ps. The dynamical processes are delayed for larger salt concentrations, from 2.5 M NaI (blue curve, empty circles) to 5 M NaI (red curve, empty circles). b) The maximum transient response scales with salt concentration, within the experimental uncertainty. c) The signal appears at longer delays when probed at 600 nm. It increases within ∼600 fs for the 2.5 M NaI aqueous sample (blue solid circles), and within ∼1 ps for the 5 M NaI solution (red solid circles). The signal is approximately constant at longer pump–probe delays, up to 20 ps. d) The signal at t_PP_ = 20 ps is proportional to the salt concentration.

Figure 2 displays the results at the probe wavelengths of ∼1000 and 600 nm. The red and blue circles correspond to 5 and 2.5 M aqueous iodide solutions, respectively. The triangles quantify the variation of OD as a function of salt concentration. For these measurements, the typical uncertainty is on the order of ±1 *mOD*.

Figure 2a reports pump–probe traces for the same optical pump pulse intensity used before in Figure 1 (100 GW/cm^2^), albeit with 100 fs pulses instead of 50 fs, and probed at λ∼1000 nm. The signals show a delay in the maximum with respect to the pump arrival time, on the time scale of ∼300–700 fs. After this delayed maximum, the signal decays within a few picoseconds. The time constants increase for the higher salt concentration. The maximum of ΔOD(t_PP_) is found at t_PP_ ∼ 0.3 ps for the 2.5 M NaI sample and at t_PP_ ∼ 0.7 ps for the 5 M solution. In Figure 2b we plot the maximum amplitude as a function of the salt concentration.

Figure 2c reports pump–probe traces at the probe wavelength λ∼600 nm. The signals slowly increase with the pump–probe delay, reach a plateau at t_PP_ ∼ 2 ps, and remain approximately constant up t_PP_ ∼ + 20 ps. The rise time is larger at higher iodide concentrations, as can be seen from the onset of the blue and red curves near t_PP_ ∼ 1 ps. In Figure 2d we plot the signal at the pump–probe delay t_PP_ = 20 ps. The transient response is proportional to the amount of dissolved salt.

**Figure 3:**
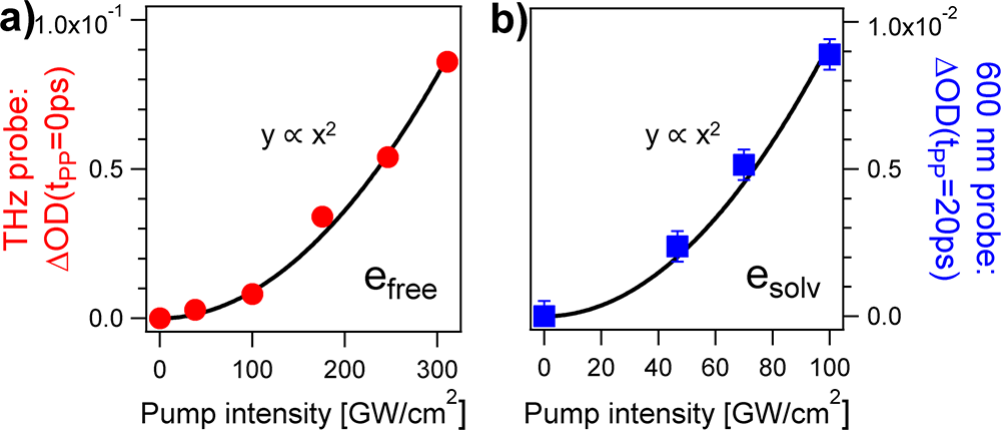
Power dependence of the transient signals of a 5 M aqueous iodide solution. a) The maximum OPTP signal at pump–probe overlap (t_PP_ = 0) is plotted as red circles. This is assigned to the delocalized or “free” electrons (e_free_). b) The blue squares correspond to the optical density measured at t_PP_ = 20 ps probed at a wavelength of 600 nm. We expect that this response depends on the amount of relaxed and solvated electrons (e_solv_). The black solid lines are quadratic fit functions of the signals vs pump intensity.

In Figure 3 we plot the transient signals as a function of pump intensity for the 5 M NaI solution. The terahertz response probed at t_PP_ = 0 is plotted in Figure 3a and corresponds to the maximum OPTP signal. The results of the optical-pump visible-probe measurements are shown in Figure 3b at the longest delay available, t_PP_ = +20 ps, where only the fully relaxed, solvated electrons contribute to the signal. The solid lines correspond to quadratic fits. Thus, the signals originate from two-photon absorption. As pure liquid water is two-photon transparent at^46^ 400 nm, the transient responses stem from the iodide anions.

It is possible to account for the main experimental results with a kinetic model whereby the delocalized electron (e_free_) transforms into the precursor (e_wet_) which, in turn, decays into the equilibrated electron (e_solv_):
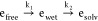
1where *k*_1_ and *k*_2_ are the rate constants.^13,47^ To a first approximation, we assume that these reactions can be described by first order processes:
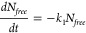
2
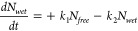
3
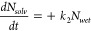
4where *N*_*free*_, *N*_*wet*_, and *N*_*solv*_ quantify the time-dependent amounts of free, wet, and solvated electrons, respectively. After time zero (t_PP_ = 0), the population of free electrons decays with rate constant *k*_1_. The number of wet electrons increases accordingly and decays with a rate equal to *k*_2_. The number of solvated electrons increases by the transformation of wet electrons. For simplicity, slower processes like geminate or nongeminate recombination involving e_solv_ are not considered.^16,48^ The solutions of the differential (eqs 2)-(4) are
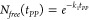
5
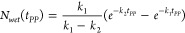
6
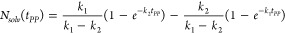
7

**Figure 4:**
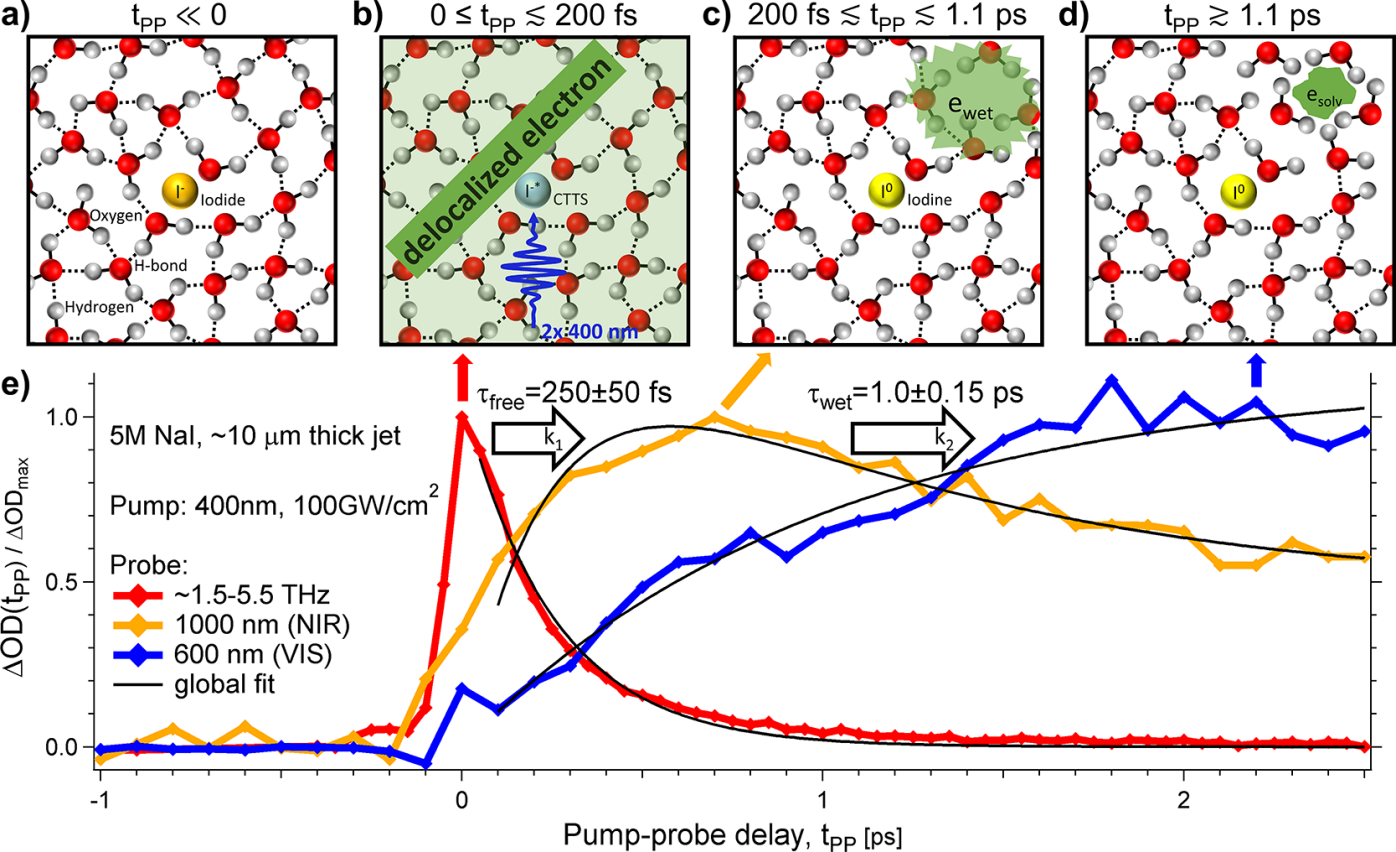
Evolution of the photoionization of aqueous iodide with two-photon absorption at 400 nm. Cartoons of microscopic salt solutions are reported in panels a), b), c), and d). The experimental results are shown in panel e). a) Sketch of the sample before the pump arrival, i.e., at t_PP_ < 0. The unperturbed liquid water molecules are displayed with oxygen in red, hydrogen in gray, and hydrogen bonds with black dashed lines. An iodide anion is represented in yellow. b) At pump–probe delays close to zero, within about 0 and t_PP_ = 200 fs, a large transient signal is probed by THz radiation (red curve in panel e). This suggests the formation of delocalized or “free” charges (e_free_). c) For t_PP_ between about 200 fs and 1.1 ps, the signal leaves the THz range and appears in the infrared (orange curve in panel e)). This can be associated to a localized precursor or “wet” electron (e_wet_). d) At longer pump–probe delays, the signal is prominently found in the visible range and the electron is solvated (e_solv_), see the blue curve in panel e). The solid black lines are obtained from the global fit procedure.

Based on previous results,^13,14,22−24,28,30,31,47,49^ we expect that the THz signal depends on *N*_*free*_, the NIR on *N*_*wet*_, and the VIS on *N*_*solv*_. However, the wet and solvated electrons have broad absorption spectra that cover the NIR and VIS frequencies with their tails.^12,28,30,47,49^ For this reason, we performed a global fit of the results in Figure 4 with the following equations:
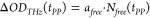
8
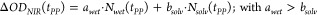
9
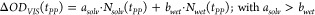
10

The resulting fit curves for t_PP_ ≥ 0.1 ps are shown with solid black lines. The best-fit parameters are *k*_1_ = (3.9 ± 0.3) · 10^12^*s*^–1^, *k*_2_ = (1.0 ± 0.2) · 10^12^*s*^–1^, *a*_*free*_ = 8.6 ± 0.5 *mOD*, *a*_*wet*_ = 17.6 ± 0.8 *mOD*, *b*_*solv*_ = 6.1 ± 0.5 *mOD*, *a*_*solv*_ = 10.8 ± 0.4 *mOD*, *b*_*wet*_ = 2.7 ± 0.4 *mOD*. The reported errors are obtained by the fit procedure.

## Discussion

The evolution of a photogenerated electron can be tracked with transient absorption as it shrinks from 20 to 40 Å (e_free_) to 4–5 Å (e_wet_) and, subsequently, to 2–3 Å (e_solv_). In a previous study on pure water,^23^ the electron was described as a particle in a potential well: the larger the well, the lower is the absorption frequency. To a first approximation, the absorption peaks in the terahertz range for the delocalized electron, in the near-infrared for the wet electron, and in the visible for the solvated electron. The rise-time and decay-time of each intermediate state determines the dynamical response at the different probe wavelengths. Based on previous results on pure water,^22−24,28,30,31,47,49^ the absorption of e_free_ should peak at THz frequencies for short pump–probe delays close to t_PP_ ∼ 0 and decay with a time constant of ∼200 fs. The absorption of e_wet_ appears at t_PP_ ∼ 100–300 fs and decays at t_PP_ ∼ 400–900 fs, for probe wavelengths in the near-infrared. The relaxed electron, e_solv_, absorbs in the visible at t_PP_ ∼ 800–2100 fs, where it reaches a plateau and remains unaffected for hundreds of picoseconds.

For the iodide solutions we observe a response similar to pure water. The instantaneous THz signal decays on a time scale comparable to the rise time in the infrared (∼200 fs) that, in turn, decays on a time scale of ∼1 ps that is comparable to the rise time in the visible range. As shown for example in Figure 1d, a large, instantaneous, and short-lived signal is found at THz frequencies, suggesting that the impulsively photogenerated electron is immediately delocalized over a large radius^22−24^ (∼20–40 Å). Such a large ejection length is consistent with the slow recombination dynamics of the solvated electron, i.e., the signal reaches a plateau and is almost identical in the probed time interval, i.e., between t_PP_ = 2 ps and t_PP_ = 20 ps in Figure 2c. For comparison, the ionization of aqueous iodide by resonant one-photon absorption is characterized by shorter ejection lengths^1,5^ (∼3–8 Å) and recombination times^3,6,8,10,12,15,16^ (15–33 ps).

As shown in Figure 1 and Figure 2, the dynamical processes are slower for higher salt concentration. This could be explained by the slower hydration dynamics: the birth of localized electronic states (e_wet_ and e_solv_) requires concerted rearrangements of hydrogen-bonded water molecules, which have slower dynamics in more viscous, concentrated ionic solution.^50−52^ The lifetime of the delocalized electron can be estimated from the relaxation time-constant of the short-lived THz response displayed in Figure 1d, which amounts to 150 ± 25 fs, 210 ± 25 fs, and 270 ± 25 fs for the 1M, 5M, and 7.5 M NaI samples, respectively.

The results are summarized in Figure 4 for the 5 M NaI sample, where cartoons of the precursor states of the solvated electron are displayed in panels a)-d) together with the transient signals detected at different probe frequencies (Figure 4e). From the kinetic model and the global fit procedure, we estimate the mean lifetime of the free electron to τ_free_ = 1/*k*_1_ = 250 ± 50 fs, and of the wet electron to τ_wet_ = 1/*k*_2_ = 1.0 ± 0.2 ps.

Bhattacharyya et al.^46^ reported the two-photon spectrum of iodide dissolved in water. Only one broad spectral feature was found in the range between ∼6 and 7 eV. By accounting for the selection rules and the position of the absorption peak with respect to the single-photon spectra, these authors argued that the two-photon band originates from ionic transitions from the 5p to the 6p orbital. Regarding our results, the two-photon transitions triggered by the ∼400 nm pulses correspond to an absorbed energy of ca. 6.2 eV. We propose that the two-photon absorption creates a delocalized Rydberg state or Wannier exciton, which results in an immediate THz response that disappears quickly and ejects electrons to large distances from the parent ions.

The pump–probe signal recorded in the VIS range on the 5 M NaI solution for a pump fluence of 100 GW/cm^2^ and at the largest delay, t_PP_ ∼ + 20 ps, is reported in Figure 2d and amounts to Δ*OD* = 8.9 ± 1.0 *mOD*. It quantifies the absorption by the photogenerated electrons after they relax and localize. If we assume that the molar extinction of a solvated electron is^53^ ε ≈ 15000 *cm*^–1^ at the probe wavelength of 600 nm, we deduce the value^54^ for the molar concentration of the photogenerated electrons, where *d* ≈ 10 μ*m* is the sample thickness. Considering the Avogadro number, this corresponds to the volume concentration *n* = 3.6 ± 0.6 · 10^17^*cm*^–3^.

In OPTP experiments on thin, free-standing, and uniformly pumped samples, the photoinduced change of the THz conductivity, σ, is described by the Tinkham formula^41,55^
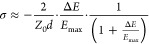
11where *Z*_0_ ≈ 376.7 Ω is the vacuum impedance and  is the relative variation of the transmitted THz field. When the pump and probe laser pulses overlap in time (t_PP_ = 0), the transient THz response is associated with the delocalized electron and reaches the maximum value  (see Figure 1c, left axis). From this quantity and eq 11, the photoinduced conductivity of the delocalized electrons amounts to . If we assume that the transient THz signal is dominated by the direct response of the delocalized electrons to the applied electromagnetic field, then the conductivity equals the product of^56^ the elementary charge (*e* ≈ 1.6 · 10^–19^*C*), carriers’ concentration (*n* = 3.6 ± 0.6 · 10^17^*cm*^–3^), and mobility. Within this approximation, we estimate that the mobility of the electrons that are impulsively generated and delocalized in liquid water amounts to . In turn, from the Nernst-Townsend-Einstein relation,^57,58^ the diffusion coefficient of the delocalized electrons is , with Boltzmann constant *k*_*B*_ and temperature *T* = 20.0 ± 0.1°*C*.

## Conclusions

We studied the photoionization of aqueous solutions of sodium iodide that was triggered by the absorption of two pump photons with a central wavelength of 400 nm. The photoionization of the aqueous 5 M NaI solution was studied with three probes: far-infrared at λ∼55–200 μm (1.5–5.5 THz), near-infrared at λ∼1 μm, and visible pulses centered at λ∼0.6 μm. The signal shifts from longer to shorter wavelengths upon increasing the pump–probe delay. Thus, we can follow the photogenerated electron from the bulk up to the fully solvated state: the free or delocalized electron decays into the wet electron within ∼250 fs, which in turn forms the solvated electron on a ∼1 ps time scale. We propose that the major contribution to the impulsive THz signal originates from a delocalized electronic state with a mean lifetime of 250 ± 50 fs and a radius of^22,24,59^ ∼20–40 Å. We suggest that such a broad electronic wave function is a frustrated Rydberg orbital of the iodide anion,^46^ a delocalized charge transfer to solvent state that is accessed with only ∼6.2 eV of optical energy. This is consistent with previous optical experiments^60^ but less so with a recent X-ray investigation.^61^ However, as these X-ray results were obtained with a pump intensity that is more than a hundred times larger than the one used here,^61^ a direct comparison could be inappropriate.

Transient terahertz spectroscopy is uniquely suited to probe the low-energy, intraband electronic transitions in condensed matter systems,^62^ and here we employ it to assess the photodetachment of electrons from iodide ions into liquid water. As a result, we provide the first direct estimate of the mobility and diffusivity of delocalized electrons in liquid water, which are approximately 500 times greater than the ones of fully hydrated electrons.^57,58^ These findings offer insights into the generation, solvation, and transport mechanisms of electrons in aqueous environments, advancing fundamental knowledge of solvated ions in the dielectric background of water.

## Data Availability

The original contributions presented in the study are included in the article, and further inquiries can be directed to the corresponding authors.
